# Usefulness of Non-High-Density Lipoprotein Cholesterol for Screening Dyslipidemia in Children and Adolescents with Overweight or Obesity: A Single-Center Retrospective Study

**DOI:** 10.3390/children12111518

**Published:** 2025-11-10

**Authors:** Hyo-Kyoung Nam, Eungu Kang, Kee-Hyoung Lee, Young-Jun Rhie

**Affiliations:** Department of Pediatrics, College of Medicine, Korea University, Seoul 02841, Republic of Korea; muguet@korea.ac.kr (H.-K.N.);

**Keywords:** cholesterol, non-high-density lipoproteins, non-fasting, dyslipidemias, adolescent, children

## Abstract

**Highlights:**

**What are the main findings?**

- Non–high-density-lipoprotein cholesterol (non-HDL cholesterol) showed high diagnostic performance for detecting elevated LDL-cholesterol, with a sensitivity of 100% in boys and 97.8% in girls.
- Random non-HDL cholesterol was non-inferior to fasting values and demonstrated significantly higher sensitivity in boys.

**What is the implication of the main finding?**

- Non-fasting non-HDL cholesterol is a practical first-line screening tool for dyslipidemia in overweight or obese children.
- More convenient screening may help identify more children at risk, allowing earlier lifestyle interventions to reduce future cardiovascular risk.

**Abstract:**

Background/Objectives: Non-high-density lipoprotein (non-HDL) cholesterol provides a practical alternative for assessing dyslipidemia, with the advantage of not requiring fasting. The aim of this study was to evaluate the utility of non-HDL cholesterol measured in random (non-fasting) samples for screening dyslipidemia in children and adolescents with overweight or obesity. Methods: We retrospectively analyzed 751 children and adolescents (268 boys and 483 girls) aged 2 to 19 years with overweight or obesity. They underwent lipid profile evaluation without fasting. Dyslipidemia was defined as the presence of one or more of the following: total cholesterol ≥ 200 mg/dL, triglycerides ≥ 100 mg/dL (ages 0–9) or ≥130 mg/dL (ages 10–19), low-density lipoprotein (LDL) cholesterol ≥ 130 mg/dL, and HDL cholesterol < 40 mg/dL. A cutoff value of ≥145 mg/dL was used for non-HDL cholesterol. The sensitivity and specificity of non-HDL cholesterol were compared with those of fasting-state data from the Korean National Health and Nutrition Examination Survey. Results: Dyslipidemia was identified in 52.6% of children and adolescents with overweight or obesity. Non-HDL cholesterol ≥ 145 mg/dL was observed in 18.7% of boys and 17.0% of girls, and dyslipidemia was observed in 94.0% and 85.4% of boys and girls with high non-HDL cholesterol, respectively. Non-HDL cholesterol demonstrated excellent performance in detecting elevated measured LDL cholesterol, with a sensitivity and specificity of 100% and 92.0%, respectively, for boys and 97.8% and 91.3%, respectively, for girls. When stratified by age, non-HDL cholesterol showed sensitivity comparable to or higher than that of total cholesterol for both boys and girls. The sensitivity of non-HDL cholesterol in random samples was significantly higher than that of non-HDL cholesterol in fasting-state samples from boys (100% vs. 94.3%, *p* = 0.010), with a similar trend shown by girls (92.9% vs. 92.3%, *p* = 0.510). Conclusions: Non-HDL cholesterol measurement may be a reliable and practical tool for dyslipidemia screening in children and adolescents with overweight or obesity. Its applicability in non-fasting conditions makes it a convenient tool for routine clinical use.

## 1. Introduction

Dyslipidemia in children and adolescents represents a significant risk factor for early atherosclerosis and cardiovascular disease in adulthood, necessitating early detection and appropriate management. Elevated serum lipid levels during childhood are considered predictors of cardiovascular complications later in life. As obesity rates continue to rise among children and adolescents [1,2], dyslipidemia has been identified in approximately 27% to 43% of those who are overweight or obese and continues into adulthood [3]. The prevalence of dyslipidemia in the overweight or obesity cohort in a previous study (28.0%) was higher than that in the general population (6.4%), with an odds ratio of 6.2 [3].

However, dyslipidemia screening can be challenging. Non-fasting lipid profiles often show elevated triglycerides, making results harder to interpret. Also, low-density lipoprotein (LDL) cholesterol is usually calculated based on triglyceride levels, which can affect accuracy [4]. Previous Korean studies using Korea National Health and Nutrition Examination Survey (KNHANES) data relied on calculated LDL cholesterol rather than direct measurements. As a result, they required fasting blood samples and focused only on adolescents aged 10 to 18 years. This study evaluated non-high-density lipoprotein (non-HDL) cholesterol measured in non-fasting samples as a practical screening tool for dyslipidemia in children and adolescents with overweight or obesity. Unlike earlier Korean studies using KNHANES data, it included children aged 2 to 19 years and compared results with directly measured LDL cholesterol.

## 2. Subjects and Methods

This retrospective study was conducted at the Department of Pediatrics in Korea University Guro Hospital from January 2015 to June 2018. We reviewed the medical records of 751 children and adolescents aged <20 years who were classified as overweight or obese according to criteria established by the Korea Centers for Disease Control and Prevention. There were 268 (35.7%) boys and 483 (64.3%) girls with a mean age was 9.6 ± 3.1 years. Obesity was defined as a body mass index (BMI) at or above the 95th percentile for age and sex, while overweight was defined as a BMI between the 85th and 95th percentiles. The reference value for the BMI percentile was based on the 2017 Korean National Growth Charts [5].

We recorded the age, sex, height, weight, and calculated BMI (kg/m^2^) by dividing weight in kilograms by height in meters squared. Height, weight, and BMI were converted into standard deviation scores (SDSs) using age- and sex-specific Korean Growth Standards. BMI percentiles were similarly derived from the same growth standards [5]. We also reviewed laboratory data including total cholesterol, triglycerides, HDL cholesterol, and directly measured LDL cholesterol, all obtained in a non-fasting state. Dyslipidemia was considered present if one or more of the following criteria were met: total cholesterol ≥ 200 mg/dL, LDL cholesterol ≥ 130 mg/dL, non-HDL cholesterol ≥ 145 mg/dL, triglycerides ≥ 100 mg/dL (age 0–9) or ≥130 mg/dL (age 10–19), and HDL cholesterol < 40 mg/dL. These criteria were based on guidelines from the National Heart, Lung, and Blood Institute (NHLBI) [6] and clinical practice guidelines for dyslipidemia in Korean children and adolescents [7].

For comparison, data were also obtained from the KNHANES conducted by the Korea Centers for Disease Control and Prevention between 2008 and 2016 [8]. This nationwide, cross-sectional survey provided anthropometric and biochemical data for fasting blood samples obtained from 6989 adolescents (3684 boys and 3305 girls) aged 10–19 years. The sensitivity of non-fasting non-HDL cholesterol was compared with that of the fasting lipid concentration in the KNHANES cohort.

All statistical analyses were performed using SPSS version 20.0 (IBM Co., Armonk, NY, USA). Continuous variables were summarized as mean ± standard deviation or median and interquartile range, whereas categorical variables were presented as number (percent). For skewed distribution of lipid parameters, values are reported as median (IQR) rather than mean ± SD. The independent *t*-test and Mann–Whitney U test were performed for comparisons between two groups. Categorical variables were compared using the chi-square test. The diagnostic performance of non-HDL cholesterol ≥145 mg/dL was assessed using McNemar’s test to compare paired binary classification outcomes, consistent with previous pediatric dyslipidemia studies, with reference to cutoff values for total cholesterol, triglycerides, and measured LDL cholesterol. Differences in the area under the receiver operating characteristic (ROC) curve (AUC) with 95% confidence intervals (CI) between total cholesterol and non-high-density-lipoprotein cholesterol were assessed. A *p*-value of <0.05 was considered statistically significant.

## 3. Results

Among the 751 participants aged 2 to 19 years with overweight or obesity, 268 (35.7%) were boys and 483 (64.3%) were girls. The mean age was 9.6 ± 3.1 years. For the 751 participants, the age distribution was similar when they were categorized into 2–9- and 10–19-year groups according to age-specific reference cutoff values. The mean SDSs for height, body weight, and BMI were 0.8 ± 1.1, 1.3 ± 1.5, and 1.6 ± 0.9, respectively. Median (IQR) lipid values were within a similar range across sex and age groups. In boys, median total cholesterol was 171 (154–189) mg/dL in those aged 2–9 years and 162 (143–185) mg/dL in those aged 10–19 years, while girls showed 169 (154–187) mg/dL and 167 (152–188) mg/dL, respectively. Corresponding median LDL cholesterol values were 99 (80–114) mg/dL and 95 (76–116) mg/dL in boys and 98 (85–112) mg/dL and 97 (82–113) mg/dL in girls. Median non-HDL cholesterol was 118 (98–134) mg/dL and 114 (92–141) mg/dL in boys and 118 (103–135) mg/dL and 117 (100–142) mg/dL in girls. Triglyceride levels were slightly higher and more variable, with medians of 102 (71–151) mg/dL and 94 (63–150) mg/dL in boys and 97 (67–143) mg/dL and 95 (69–140) mg/dL in girls across the same age strata. Median HDL cholesterol ranged from 46–62 mg/dL across groups. The overall prevalence of dyslipidemia was 52.6%. Elevated non-HDL cholesterol (≥145 mg/dL) was observed in 18.7% boys and 17.0% girls, and dyslipidemia was observed in 94.0% boys and 85.4% girls with high non-HDL cholesterol, respectively. The proportions of children with elevated total cholesterol, triglycerides, and measured LDL cholesterol were 16.0%, 35.4%, and 11.6%, respectively, among boys and 14.0%, 32.6%, and 10.1%, respectively, among girls (Table 1).

With regard to diagnostic performance, non-HDL cholesterol ≥145 mg/dL showed high sensitivity and specificity for the detection of elevated measured LDL cholesterol, exhibiting 100% sensitivity and 92.0% specificity for boys and 97.8% sensitivity and 91.3% specificity for girls (*p* < 0.001 for both; Table 2). For the identification of high total cholesterol, sensitivity and specificity values were 86.0% and 94.2% for boys and 79.0% and 92.2% for girls (*p* < 0.001 for both). For the detection of elevated triglycerides, sensitivity and specificity values were 23.1% and 84.4%, respectively, for boys and 15.9% and 82.1%, respectively, for girls (*p* = 0.161 and *p* = 0.364, respectively).

As a tool for detecting high LDL cholesterol, total cholesterol showed sensitivity and specificity values of 93.5% and 94.1%, respectively, for boys and 88.9% and 95.0%, respectively, for girls across all age groups. Although non-HDL cholesterol demonstrated a slightly higher sensitivity than did total cholesterol for both boys and girls, the differences were not statistically significant (boys: 100% vs. 93.5%, *p* = 0.753; girls: 97.8% vs. 88.9%, *p* = 0.460).

When stratified by age, non-fasting non-HDL cholesterol showed a significantly higher detection rate than did total cholesterol for girls aged 2–9 years (*p* = 0.031), whereas it showed a detection rate comparable to that of total cholesterol for boys aged 2–9 years. For boys aged 10–19 years, non-fasting non-HDL cholesterol showed slightly lower specificity (90.3% vs. 96.3%); however, its superior sensitivity resulted in a significantly higher detection rate (*p* = 0.010). For girls aged 10–19 years, both non-HDL cholesterol and total cholesterol demonstrated high diagnostic accuracy for the detection of elevated LDL cholesterol, with no statistically significant difference (*p* = 0.013; Table 3).

When non-fasting non-HDL cholesterol levels were compared with the levels in fasting samples from KNHANES, the former showed slightly lower specificity (90.3% vs. 95.7%) but significantly higher sensitivity for the detection of high LDL cholesterol in boys (100% vs. 94.3%, *p* = 0.010). For girls, both sensitivity (92.9% vs. 92.3%) and specificity values (92.0% vs. 96.5%) were comparable, with no statistically significant difference (*p* = 0.510; Table 4).

**Figure 1:**
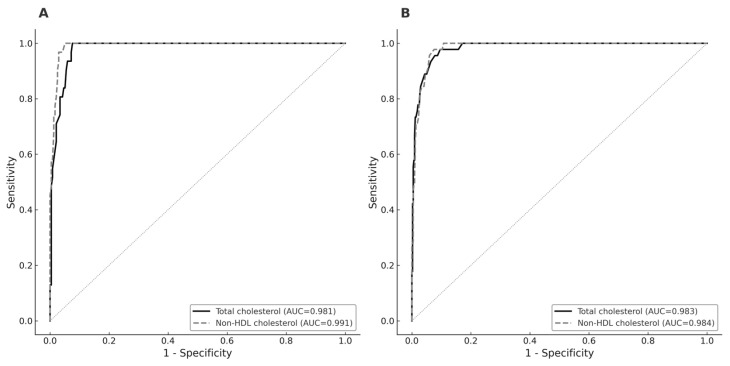
Receiver operating characteristic curve (ROC) comparing the diagnostic performance of total cholesterol (solid black line) and non-high-density-lipoprotein cholesterol (dashed gray line) for detecting LDL-cholesterol level ≥130 mg/dL. Panel (**A**) shows results for boys, and Panel (**B**) shows results for girls. The area under the ROC curve (AUC) values are presented with 95% CI.

In ROC curve analysis, both total cholesterol and non-HDL cholesterol showed a similar diagnostic performance for identifying LDL cholesterol level ≥130 mg/dL. In boys, AUCs for total cholesterol and non-HDL cholesterol was 0.981 (95% CI: 0.965–0.993) and 0.991 (95% CI: 0.981–0.998) respectively (*p* = 0.656). In girls, AUCs were for total cholesterol and non-HDL cholesterol was 0.983 (95% CI: 0.971–0.994) and 0.984 (95% CI: 0.973–0.992), respectively (*p* = 0.982) (Figure 1).

## 4. Discussion

This retrospective study demonstrated that approximately half of the included children and adolescents classified as overweight or obese exhibited dyslipidemia, as defined by lipid profile criteria in the NHLBI guidelines. Our findings highlighted the clinical utility of non-HDL cholesterol, particularly for the identification of elevated directly measured LDL cholesterol. Furthermore, the diagnostic sensitivity of non-HDL cholesterol measured in random samples was comparable to or higher than that of fasting non-HDL cholesterol or total cholesterol in both boys and girls.

Dyslipidemia during childhood, especially in the presence of obesity, remains a growing public health issue because of its strong correlation with future cardiovascular morbidity and mortality. Early detection of atherosclerotic changes in children and adolescents is important, as the condition often persists in adulthood and significantly increases cardiovascular risk later in life [9,10]. Recent data indicate an increasing trend in dyslipidemia prevalence among Korean children and adolescents. The prevalence of dyslipidemia increased from 19.7% (KNHANES 2007–2009) to 39.5% (KNHANES 2016–2018) in boys and from 29.7% to 39.5% [11,12] in girls. Among Korean adolescents aged 10–19 years, approximately 20% have at least one lipid abnormality, and over half (56.1%) of adolescents with obesity are affected by dyslipidemia. In the present study, the dyslipidemia prevalence of 52.6% among children and adolescents with overweight or obesity was comparable with the recent national estimates reported in KNHANES; this further supports the relevance of routine lipid screening in this at-risk population.

Among children and adolescents with dyslipidemia, 77.1% received nutrition and physical activity counseling, and 57.1% were referred to a dietitian. However, only 68.6% attended follow-up visits, and just 31.4% had repeat lipid testing. These results highlight the need for more practical and widely applicable screening strategies [13].

Over the past decades, universal screening has been emphasized, as targeted screening alone fails to detect a large proportion of children and adolescents with dyslipidemia including those with genetic lipid disorders [6,14]. In 2011, the U.S. NHLBI Expert Panel revised cutoff values for children based on the population distribution and recommended additional universal screening for all children and adolescents at ages 9–11 years and again at 17–21 years [6]. The National Lipid Association also recommended universal screening with a fasting lipid profile [15].

Despite the recommendations for universal lipid screening, current screening rates remain suboptimal. Previous studies have shown that only one-third of children aged 9–11 years undergo lipid screening, partly because NHLBI/AAP guidelines require a fasting lipid profile and retesting within approximately 3 months. In addition, the recommendation to measure LDL cholesterol has further limited adherence in clinical practice [15].

LDL cholesterol measurement does not account for all classes of atherogenic lipoproteins, may underestimate low-concentration lipoproteins, and requires overnight fasting. The non-HDL cholesterol level is calculated by subtracting HDL cholesterol from total cholesterol levels, and it encompasses all atherogenic apolipoprotein B-containing lipoproteins, including very-low-density lipoproteins (VLDL), intermediate-density lipoproteins (IDL), LDL, lipoprotein(a) [Lp(a)], and chylomicron remnants.

Non-HDL cholesterol has been associated with cardiovascular morbidity and mortality and has emerged as a strong predictor of cardiovascular outcomes in adults [16]. Non-HDL cholesterol levels in children and adolescents have been found to be associated with the risk of cardiovascular disease in adulthood. Furthermore, non-HDL cholesterol is also a useful marker of dyslipidemia in children and adolescents with overweight or obesity and offers several advantages over LDL cholesterol in the prediction of adult dyslipidemia, carotid intima–media thickness, and non-lipid cardiovascular risk factors [17,18]. Children with discordantly high non-HDL cholesterol but normal LDL cholesterol were found to exhibit an approximately two-fold higher risk of atherosclerotic cardiovascular outcomes in adulthood [19]. Thus, non-HDL cholesterol is useful because it offers a broader atherogenic risk assessment than does LDL cholesterol alone [20].

In this study, non-HDL cholesterol showed 100% sensitivity for detecting high LDL cholesterol levels in both boys and girls aged 2–9 years, while its specificity was comparable to that of total cholesterol (91–95%). Although non-HDL cholesterol showed slightly lower specificity than did total cholesterol (90.3–92.0% vs. 96.3–97.3%) for both boys and girls aged 10–19 years, its sensitivity resulted in a comparable or higher detection rate (92.9–100% vs. 88.2–100%). Although the data are retrospective, both indices were measured simultaneously in each individual, minimizing concerns regarding misclassification. As direct fasting data for the same participants were unavailable, we indirectly compared non-fasting-state data obtained from Korean national data collected for individuals aged 10–19 years. The results revealed that the diagnostic sensitivity of non-HDL cholesterol was higher in the non-fasting state than in the fasting state. Given its high sensitivity and perfect negative predictive value, non-HDL cholesterol may serve as a non-inferior, more practical, and reliable indicator for screening LDL cholesterol-related dyslipidemia, even in the non-fasting state.

Non-HDL cholesterol is easy to obtain and cost-effective. Because it is calculated as total cholesterol minus HDL-cholesterol, it can be measured in the non-fasting state without requiring triglyceride values; however, triglyceride measurement remains necessary when specifically evaluating hypertriglyceridemia. Testing without fasting enhances patient convenience and improves adherence to recommended screening protocols. These methodological benefits enhance its clinical utility for children and adolescents.

The AAP and National Lipid Association endorse non-HDL cholesterol measurement as a preferred secondary screening method after an initial total cholesterol or LDL cholesterol assessment [21]. Similar guidelines have been adopted in Korea, where universal screening using non-HDL cholesterol measurement in the non-fasting state is recommended for all children and adolescents aged 9–11 and 17–21 years [9,22].

Several studies have not demonstrated sufficient evidence regarding the benefits of lipid screening in children and adolescents aged <20 years [23,24]. However, multiple studies have shown that diagnosing dyslipidemia (in particular high non-HDL cholesterol levels) and treating it from childhood has led to a reduction in cardiovascular mortality in adulthood [17,25,26].

Despite growing evidence, there is a lack of universally accepted pediatric thresholds for non-HDL cholesterol levels. Moreover, there is little evidence of variability in non-HDL cholesterol thresholds between fasting and non-fasting blood samples. Longitudinal intervention studies should examine how non-HDL cholesterol-guided screening affects later outcomes.

This study has limitations that should be considered when interpreting the findings. First, its retrospective and single-center design may introduce selection bias and limit the generalizability of the findings to broader pediatric populations. Second, the use of a single lipid measurement for each participant may be susceptible to biological variability and transient influences such as pubertal stage, dietary intake, or physical activity, potentially affecting the accuracy of dyslipidemia classification. Third, although national survey data were used for indirect comparisons, differences in study populations, data collection methods, and temporal factors may limit the validity of cross-study comparisons. Therefore, the results should be interpreted with caution, and future multicenter prospective studies with repeated lipid assessments are warranted to validate and expand upon our findings.

## 5. Conclusions

In conclusion, non-HDL cholesterol measurement may be a reliable tool for screening dyslipidemia in children and adolescents with overweight or obesity and offers practical benefits over LDL cholesterol measurement. One of its key advantages lies in its utility in the non-fasting state, making it more feasible for routine clinical screening. The incorporation of non-HDL cholesterol measurement into standard pediatric lipid screening protocols may contribute to improved early detection and intervention, thereby reducing the burden associated with pediatric obesity and dyslipidemia. Future clinical guidelines should aim to focus on more effective screening strategies to enhance adherence and health outcomes in children and adolescents.

## Figures and Tables

**Table 1:** Clinical and biochemical characteristics of participants and the prevalence of dyslipidemia.

	Boys	Girls	Total
Number (%)	268 (35.7%)	483 (64.3%)	751 (100%)
Age			
Subgroup 2–9 years	8.0 (6.0–9.0)	8.0 (7.0–8.0)	8.0 (7.0–9.0)
Subgroup 10–19 years	12.0 (11.0–15.0)	11.0 (10.0–14.0)	12.0 (10.0–15.0)
Body mass index SDS	1.6 (1.1–2.2)	1.7 (1.2–2.1)	1.7 (1.2–2.2)
Total cholesterol ≥ 200 mg/dL (%)	43 (16.0%)	62 (12.8%)	105 (14.0%)
Non-HDL cholesterol ≥ 145 mg/dL (%)	50 (18.7%)	82 (17.0%)	132 (17.6%)
Measured LDL cholesterol ≥ 130 mg/dL (%)	31 (11.6%)	45 (9.3%)	76 (10.1%)
Triglycerides ≥ 100 or 130 mg/dL ^a^ (%)	95 (35.4%)	150 (31.1%)	245 (32.6%)
HDL cholesterol < 40 mg/dL (%)	56 (20.9%)	74 (15.3%)	130 (17.3%)

Data are expressed as median (IQR) or number (%). ^a^ Triglycerides ≥ 100 mg/dL at age 0–9 years and ≥130 mg/dL at age 10–19 years. SDS = standard deviation score, HDL = high-density lipoprotein, LDL = low-density lipoprotein.

**Table 2:** Diagnostic performance of non-HDL cholesterol for the detection of dyslipidemia in boys and girls.

Criteria	Cholesterol Cutoff Values	Number (%) *	Sensitivity	Specificity
Boys	Total cholesterol ≥ 200 mg/dL	43 (16.0%)	86.0 (72.7–93.4)	94.2 (90.4–96.6)
	Triglycerides ≥ 100 or 130 mg/dL ^a^	108 (40.3%)	23.1 (16.2–31.9)	84.4 (78.0–89.2)
	Measured LDL cholesterol ≥ 130 mg/dL	31 (11.6%)	100.0 (89.0–100.0)	92.0 (87.8–94.8)
Girls	Total cholesterol ≥ 200 mg/dL	62 (12.8%)	79.0 (67.4–87.3)	92.2 (89.2–94.4)
	Triglycerides ≥ 100 or 130 mg/dL ^a^	220 (45.5%)	15.9 (11.7–21.3)	82.1 (77.0–86.3)
	Measured LDL cholesterol ≥ 130 mg/dL	45 (9.3%)	97.8 (88.4–99.6)	91.3 (88.3–93.6)

* Data are expressed as number (%) or % (95% CI). ^a^ Triglycerides ≥ 100 mg/dL at age 0–9 years and ≥130 mg/dL at age 10–19 years.

**Table 3:** Diagnostic performance of non-fasting non-HDL cholesterol for the detection of high LDL cholesterol in boys and girls stratified by age.

Criteria	Cholesterol Cutoff Values	Sex	Sensitivity (%)	Specificity (%)
Age (2–9 years)	Total cholesterol ≥ 200 mg/dL	Boys	100.0 (78.5–100.0)	91.3 (84.2–95.3)
		Girls	83.9 (67.4–92.9)	94.5 (91.6–96.4)
	Non-HDL cholesterol ≥ 145 mg/dL	Boys	100.0 (78.5–100.0)	94.2 (87.9–97.3)
		Girls	100.0 (89.0–100.0)	91.2 (87.8–93.7)
Age (10–19 years)	Total cholesterol ≥ 200 mg/dL	Boys	88.2 (65.7–96.7)	96.3 (91.6–98.4)
		Girls	100.0 (78.5–100.0)	97.3 (90.8–99.3)
	Non-HDL cholesterol ≥ 145 mg/dL	Boys	100.0 (81.6–100.0)	90.3 (84.1–94.2)
		Girls	92.9 (68.5–98.7)	92.0 (83.6–96.3)

Data are expressed as number (%) or % (95% CI).

**Table 4:** Comparison of the diagnostic performance of non-HDL cholesterol for the detection of high LDL cholesterol between fasting and non-fasting states in boys and girls.

Criteria	Cholesterol Cutoff Values	Sensitivity (%)	Specificity (%)
Boys	Non-HDL cholesterol in non-fasting state	100	90.3
	Non-HDL cholesterol in fasting state	94.3	95.7
Girls	Non-HDL cholesterol in non-fasting state	92.9	92.0
	Non-HDL cholesterol in fasting state	92.3	96.5

Data are expressed as number (%).

## Data Availability

All relevant data are included in the paper. Otherwise, the raw data analyzed during the current study are not publicly available as data contain sensitive patient information. Anonymized data not included in this article will be shared from the corresponding author on reasonable request by qualified investigator.
